# Pharmacological Effects and Underlying Mechanisms of Licorice-Derived Flavonoids

**DOI:** 10.1155/2022/9523071

**Published:** 2022-01-17

**Authors:** Yufan Wu, Zhuxian Wang, Qunqun Du, Zhaoming Zhu, Tingting Chen, Yaqi Xue, Yuan Wang, Quanfu Zeng, Chunyan Shen, Cuiping Jiang, Li Liu, Hongxia Zhu, Qiang Liu

**Affiliations:** ^1^School of Traditional Chinese Medicine, Southern Medical University, Guangzhou 510515, China; ^2^Integrated Hospital of Traditional Chinese Medicine, Southern Medical University, Guangzhou 510315, China

## Abstract

*Glycyrrhizae Radix et Rhizoma* is the most frequently prescribed natural medicine in China and has been used for more than 2,000 years. The flavonoids of licorice have garnered considerable attention in recent decades due to their structural diversity and myriad pharmacological effects, especially as novel therapeutic agents against inflammation and cancer. Although many articles have been published to summarize different pharmacological activities of licorice in recent years, the systematic summary for flavonoid components is not comprehensive. Therefore, in this review, we summarized the pharmacological and mechanistic data from recent researches on licorice flavonoids and their bioactive components.

## 1. Introduction

*Glycyrrhizae Radix et Rhizoma* is the most frequently prescribed natural medicine in China and has been widely used for more than 2,000 years. The genus *Glycyrrhiza* is composed of approximately 30 species [1], of which *G. inflate* Bat., *G. uralensis* Fisch., and *G. glabra* L. are the origins of licorice according to the pharmacopeia of the People's Republic of China [2]. As it does not represent a hazard to the public, it is widely used in food, tobacco, and cosmetics as condiments and ingredients [3]. As a herbal medicine, *G. Radix et Rhizoma* is mainly used to treat respiratory and gastrointestinal symptoms and to quench thirst during fasting [4]. Furthermore, it is also prescribed as part of both holistic and mainstream medicine for various diseases, which can be attributed to its extensive pharmacological activities including anti-inflammatory, anticancer, antioxidant, antidiabetic, antiulcer, antiallergy, and antiviral effects [5, 6].

**Figure 1:**
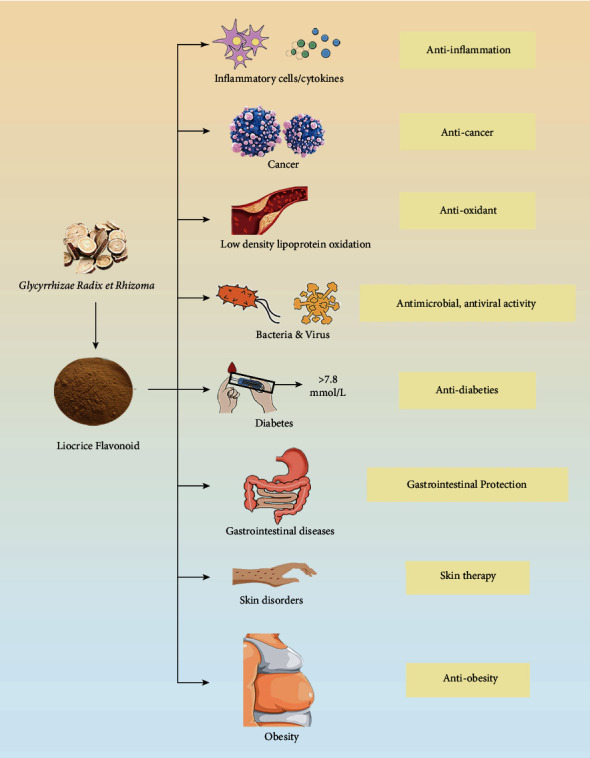
Pharmacological activities of licorice flavonoids.

**Figure 2:**
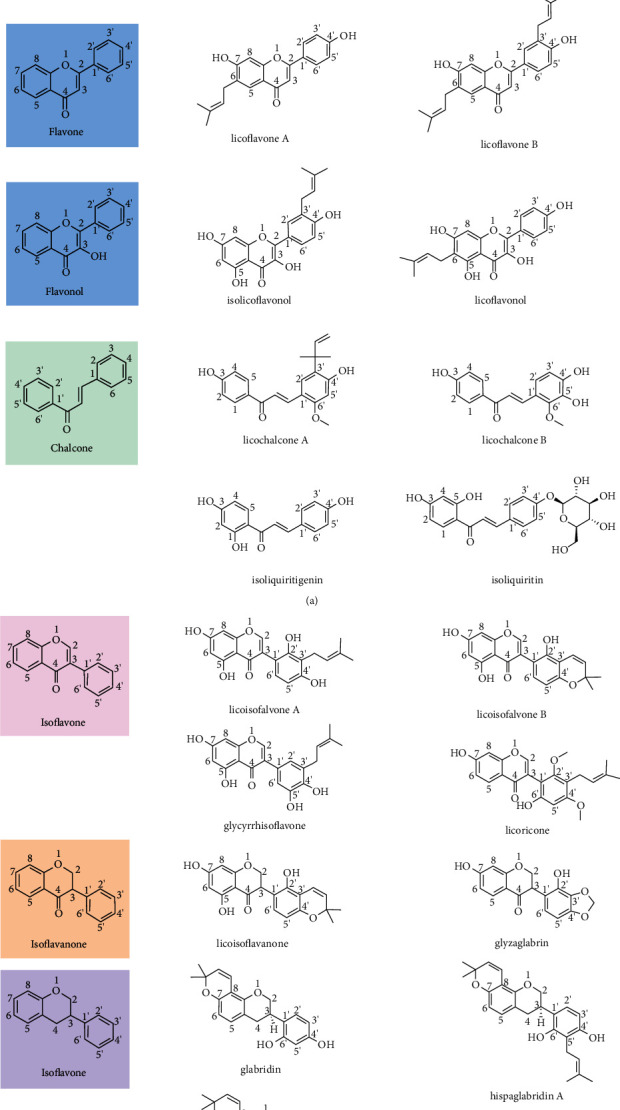
(a) Flavanone, flavone, flavonol, and chalcone structure from licorice. (b) Isoflavone, isoflavanone, isoflavan, and isoflavene structure from licorice.

Over 400 compounds have been identified in licorice, including triterpene saponins, flavonoids [7], coumarins, phenolics, pterocarpan, and others [8]. In addition, 300 flavonoids with a basic C_6_-C_3_-C_6_ skeleton derived from licorice are currently known, including flavanones, flavones, flavonols, chalcones, isoflavones, isoflavanones, isoflavans, and isoflavenes [9–11], which have considerable structural diversity [5] and exhibit anti-inflammatory, antioxidant [12], antitumor [13], antibacterial, antiviral [14–16], gastroprotective [17], and other effects (shown in Figure 1). For instance, licochalcone A inhibits the growth and metastasis of colonic tumors by downregulating inflammatory mediators and modifying the tumor microenvironment [18]. The chemical structures and main components of licorice flavonoids are summarized in Figure 2.

There is a substantial body of research on the biological activities, molecular and cellular mechanisms, and the active components of licorice flavonoids. Although many articles have been published to summarize different pharmacological activities of licorice in recent years, most of them tended to focus on triterpenoid components or one aspect of the effect, and the systematic summary for flavonoid components is not comprehensive. The purpose of this review is to summarize the pharmacological effects and mechanisms of action from recent researches on licorice flavonoids and their bioactive components.

## 2. Anti-Inflammation

Inflammation is the protective response to harmful stimuli such as mechanical injury, pathogens, damaged cells, or other irritants and involves local blood vessels, immune cells, and molecular factors. The inflammatory response restricts and eliminates invading pathogens, removes and/or absorbs necrotic tissues and cells, and repairs injured tissues. Based on the clinical course and predominant cell types, it can be classified as acute or chronic. Acute inflammation is mediated by the rapid infiltration of granulocytes into the affected tissues and has a finite duration, whereas chronic inflammation is a prolonged condition induced by the direct infiltration of mononuclear immune cells like macrophages, monocytes, lymphocytes, and so forth [19]. At the molecular level, the inflammatory response is mediated by cytokines such as interleukin-1*β* (IL-1*β*) and tumor necrosis factor-*α* (TNF-*α*), vascular endothelial growth factor (VEGF), nitric oxide (NO), prostaglandin (PG), and leukotriene (LT) [20–22], along with the nuclear factor kappa B (NF-*κ*B) [23, 24], Janus kinase/signal transducers and activators of transcription (JAK/STAT) [25], Nrf2/Keap1/ARE [19], and toll-like receptors (TLRs) pathways [26].

### 2.1. Effect on Inflammatory Diseases

The flavonoid structure is endowed with excellent anti-inflammatory property; for instance, viscosine, a flavonoid from *Dodonaea viscosa* showed anti-inflammatory and antipyretic properties as it reduced the concentration of PGE2 in brain through its mPGES-1 inhibitory action [27]. In the same way, licorice flavonoids display favorable anti-inflammatory effect and have shown therapeutic effects in pneumonia, hepatitis, ulcerative colitis, gastritis, and other inflammatory diseases [28]. Total flavonoids (TFF) from *G. uralensis* alleviated localized inflammation in the carrageenan-stimulated rat paw edema model and dimethylbenzene (DMB) induced ear vasodilatation assay in a dose-dependent manner [29]. Likewise, licorice flavonoids mitigated the acute pulmonary inflammation induced by intratracheal administration of lipopolysaccharides (LPS) at the doses of 3, 10, and 300 mg/kg, as indicated by reduced infiltration of macrophages, lymphocytes, and especially neutrophils in the accumulated bronchoalveolar lavage fluid (BALF) [30].

The main anti-inflammatory active flavonoids of licorice include the chalcones like licochalcone A and licochalcone B, and isoliquiritigenin, isoflavones such as isoangustone A, and isoflavans such as glabridin and licoricidin [31]. Licochalcone A, licochalcone B, 5-(1,1-dimethylallyl)-3,4,40-trihydroxy-2-methoxychalcone, and echinatin suppressed the LPS-induced production of reactive oxygen species (ROS) in RAW 264.7 macrophages in a dose-dependent manner and downregulated the levels of prostaglandin E2 (PGE2), IL-6, and NO in LPS-stimulated macrophages [32]. Moreover, isoliquiritigenin mitigated high-fat-diet-induced inflammation in a mouse model by significantly reducing the infiltration of inflammatory cells into the white adipose tissue of epididymis (eWAT) [33]. Glabridin also exhibited an anti-inflammatory effect against diabetes-related vascular dysfunction by downregulating LPS-induced NO production, as well as the expression of inducible nitric oxide synthase (iNOS) gene under high-glucose conditions [34].

*In vitro* and *in vivo* studies on the anti-inflammatory effects of licorice flavonoids are summarized in Tables 1 and 2, respectively.

### 2.2. Mechanism Underlying Anti-Inflammatory Effect

**Figure 3:**
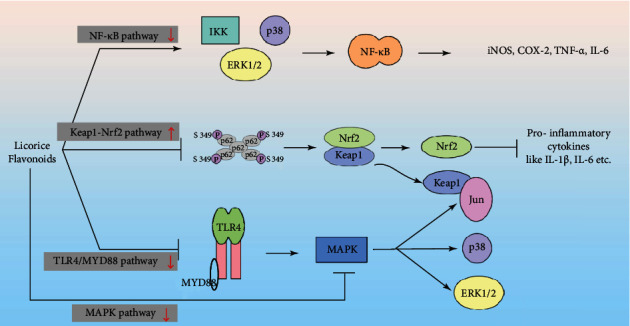
The main signaling pathway of anti-inflammation of licorice flavonoids.

The inflammatory process is highly synchronized and progresses sequentially through cell migration and infiltration, enzyme activation, fluid extravasation, inflammatory mediator release, tissue breakdown, and repair [55]. The mechanisms, factors, and pathways that induce and exacerbate inflammation are highly complex, and licorice flavonoids restrain the inflammatory mediators and cytokines by targeting multiple immune-related pathways (Figure 3).

#### 2.2.1. NF-*κ*B Signaling Pathway

The NF-*κ*B axis is the representative proinflammatory signaling pathway, and the activation of the transcription factor NF-*κ*B culminates in the expression of genes encoding proinflammatory cytokines like TNF-*α* and IL-1 [24], adhesion molecules, chemokines, COX-2, MMPs, and iNOS [56]. Given the crucial role of NF-*κ*B in the pathogenesis of inflammation, its blockade is a rational therapeutic strategy against various chronic inflammatory conditions.

Isoliquiritigenin exerts its anti-inflammatory effects by suppressing NF-*κ*B activity, consequently leading to a decrease in the levels of TNF-*α*, IL-6, IL-1*β*, and IL-8 and other proinflammatory factors [57, 58]. Another study showed that isoliquiritigenin inhibited NF-*κ*B and the downstream iNOS, TNF-*α*, COX-2, and IL-6 in RAW 264.7 cells *via* downregulation of extracellular signal-regulated kinase 1/2 (ERK1/2), nuclear factor kappa B kinase (IKK), and p38 phosphorylation [59]. Likewise, licochalcone A, licochalcone D, and licochalcone B significantly inhibited LPS-induced transcriptional activation of NF-*κ*B and phosphorylation at serine 276 by suppressing protein kinase A (PKA) [60]. In addition, licochalcone E inhibited the nuclear translocation of NF-*κ*B and decreased the levels of its multiple downstream targets such as iNOS, vascular cell adhesion molecule-1 (VCAM-1), and intercellular adhesion molecule-1 (ICAM-1) in LPS-stimulated H9c2 cells [61]. Licochalcone E also ameliorated chronic allergic contact dermatitis and inhibited the production of IL-12p40 in a dose-dependent manner by downregulating NF-*κ*B, indicating its therapeutic potential in skin inflammatory disorders [62].

#### 2.2.2. Nuclear Factor-Erythroid 2 Related Factor 2 (Nrf2) Signaling Pathway

A recent study showed that licochalcone A alleviated the symptoms of arthritis by suppressing the proliferation of the inflammatory cells. Mechanistically, licochalcone A slowed cell cycle transition and enhanced apoptosis, inhibited proinflammatory cytokine secretion, and upregulated antioxidant enzyme expression by activating the Keap1-Nrf2 signaling pathway. It promoted Nrf2 accumulation and nuclear translocation and increased p62 phosphorylation [63]. Isoliquiritigenin protected against cigarette-smoke-induced chronic obstructive pulmonary disease (COPD) via suppression of inflammation and oxidative stress by targeting the Nrf2 and NF-*κ*B signaling pathways [64]. In addition, isoliquiritigenin also downregulated NADPH oxidase 2 (NOX2) and NOX4 levels, promoted the dissociation of Keap1 and Nrf2, and activated the NAD(P)H quinone dehydrogenase 1 (NQO1), heme oxygenase-1 (HO-1), glutamate-cysteine ligase (GCLC), and GCLM genes [65]. Another study showed that isoliquiritin can activate Nrf2 signaling in murine macrophages by suppressing Keap1 and increasing Nrf2 translocation and increasing the expression levels of UGT1A1, HO-1, and NQO1, eventually suppressing the inflammatory responses [66]. Taken together, licorice flavonoids exert a potent anti-inflammatory effect through Nrf2 pathway activation.

#### 2.2.3. Other Signaling Pathways

As discussed in Section 2.2.2, the anti-inflammatory effects of licorice flavonoids are driven by complex mechanisms and multiple signaling pathways. Isoliquiritigenin mitigated DSS-induced colitis in a mouse model by inhibiting the mitogen-activated protein kinase (MAPK) signaling pathway via suppression of ERK1/2 and p38 phosphorylation. Likewise, liquiritin also targeted the MAPK pathway in rheumatoid arthritis (RA) by downregulating the B-cell lymphoma-2 (Bcl-2)/Bcl-2-associated X (Bax) ratio, c-Jun N-terminal kinase (JNK), and p38 phosphorylation, as well as VEGF expression [67, 68]. Moreover, isoliquiritigenin significantly decreased the levels of toll-like receptor 4 (TLR4) protein and its downstream targets including myeloid differentiation primary response 88 (MYD88), a phosphorylated inhibitor of nuclear factor kappa B (p-I*κ*B*α*), and p-NF-*κ*B. Isoliquiritigenin-mediated blockade of the TLR4/MYD88 pathway had neuroprotective and anti-inflammatory effects in a kainic acid (KA) stimulated model of epileptogenesis [69]. Zhu et al. found that isoliquiritigenin inhibited receptor activator of nuclear factor-*κ*B ligand (RANKL) stimulated osteoclastogenesis and inflammatory bone loss by inhibiting receptor activator of I*κ*B*α*/NF-*κ*B, MAPK, nuclear factor-*κ*B-TNF receptor-associated factor 6 (RANK-TRAF6), and activator protein-1(AP-1) signaling pathways [70]. Licochalcone A blocked the induction of caspase-1 and IL-1*β* in human SZ95 sebocytes and primary mouse macrophages infected with *Propionibacterium acnes* and controlled *P. acnes*-induced skin inflammation by targeting NOD-, LRR-, and pyrin-domain-containing protein 3 (NLRP3) inflammasome [71]. Total flavonoids of *Radix Glycyrrhiza* inhibited the LPS/IFN-*γ*-induced inflammatory response in RAW 264.7 macrophages by inhibiting iNOS expression via the ERK/NF-*κ*B/miR-155 pathway [72].

## 3. Anticancer

### 3.1. Anticancer Effect

Cancer results from human cells that slip from reining, having been recruited and to some extent transformed into pathological organisms or building the block of tumor [73]. Licorice flavonoids have established anticancer effects, and the underlying mechanisms are diverse. For instance, 70% ethanol-extracted total flavonoids markedly reduced tumor mass of breast cancer cell MDA-MB-231 xenografts by suppressing iNOS expression [72]. In addition, licochalcone A inhibited the growth and proliferation of HepG2 cells by blocking the MAPK signaling pathway [74]. Furthermore, several licorice flavonoids have exhibited proapoptotic [75–77] and antimetastatic [18, 78] effects in diverse cancer cell lines and animal models. In this section, we have summarized the effects and underlying mechanisms of licorice flavonoids on solid tumors and cancer cells.

#### 3.1.1. Hepatocellular Carcinoma

Studies have demonstrated the anti-liver-cancer effects of glabridin [79], licochalcone A [80], licochalcone B [81], and isoliquiritigenin [78]. Glabridin significantly blocked cell proliferation in Huh7 human hepatoma cells and induced apoptosis through poly ADP-ribose polymerase (PARP) cleavage, caspase-3, caspase-8, and caspase-9 activation and increased microtubule-associated protein 1 light chain 3-II (LC3-II) and Beclin-1 protein expression [76]. Moreover, it restrained the migration and invasion of hepatocellular carcinoma cells and effectively prevented the formation of hepatoma xenografts in a mouse model [79]. Recent studies showed that isoliquiritigenin inhibited the proliferation, migration, and metastasis of Hep3B human liver cancer cells and exhibited cytotoxic effects on HepG2 and Hep3B cells, indicating that it can block hepatocellular carcinoma genesis and metastasis [78, 82]. The antihepatocellular carcinoma effect of liquiritigenin was embodied in enhancing apoptotic rate, inhibiting cell viability, and inducing overrelease of lactate dehydrogenase and up-regulated intracellular ROS level and caspase 3 activity in HepG2 and PLC/PRL/5 cells [83]. Both licochalcone A and licochalcone B blocked the growth of HepG2 cells via terminating cell cycle at G2/M phase and induced apoptosis by modulating the expression of genes involved in cell cycles [80, 81].

#### 3.1.2. Lung Cancer

Lung cancer is one of the most prevalent malignancies and is associated with a poor prognosis. Despite advances in chemotherapy over the past two decades, the survival rates of patients are still dismal. Studies show that flavonoids isolated from *Glycyrrhiza*, such as liquiritin, isoliquiritigenin [84], and licochalcones [85], can effectively control lung cancer progression. Licochalcone A induced apoptosis in non-small cell lung cancer (NSCLC) cells by promoting autophagy and simultaneously enhancing the expression of the endoplasmic reticulum stress-related mediator C/EBP homologous protein (CHOP), which is known to clear damaged cells by triggering both apoptotic and autophagic pathways [85]. In addition, it suppressed cell growth and induced apoptosis in A549 and H460 NSCLC cell lines [86]. Licochalcone B and licochalcone D displayed proapoptotic and antiproliferative effects in epidermal growth factor receptor (EGFR) mutant NSCLC cell line HCC827 via caspases activation, PARP cleavage, and relevant proteins modulation [13, 87]. Furthermore, glabridin exhibited suppression of cell metastasis by deterring migration and invasion of A549 cells and decreasing A549-mediated angiogenesis both *in vitro* and *in vivo* [88]. Echinatin restrained gefitinib-sensitive/resistant NSCLC cells by inhibiting cell multiplication and inducing ROS production in EGFR mutant NSCLC cell line HCC827 and human lung epithelial cell line NL20 [89]. Finally, the combination of liquiritin, isoliquiritigenin, and isoliquiritin induced apoptosis in the A549 NSCLC cell line by inhibiting the p53-dependent pathway and also affecting the downstream targets of Akt [84].

#### 3.1.3. Gastric Cancer

Several licorice flavonoids including licochalcone A [90], liquiritin [91], and liquiritigenin [92] have shown therapeutic effects against gastric cancer, of which licochalcone A shows the highest cytotoxicity in gastric cancer cells [90]. In normal cells, glucose is metabolized into H_2_O and CO_2_ that generate ATP to meet the energy requirements. However, tumor cells largely depend on aerobic glycolysis, wherein glucose is converted to pyruvate and lactate, for their energy needs. This phenomenon is known as the Warburg effect and results in the accumulation of lactate which creates a highly acidic tumor microenvironment, leading to enhanced chemoresistance, migration, and metastasis of the tumor cells [93, 94]. Licochalcone A suppressed hexokinase 2 (HK2) induced glycolysis in the human gastric BGC-823 cells, which not only inhibited proliferation and clonogenic survival [95] but also induced apoptosis in the tumor cells [96]. Furthermore, liquiritin monotherapy moderately inhibited the proliferation and migration of cisplatin (DDP) resistant [91] or TNF-related apoptosis-inducing ligand (TRAIL) resistant [97] gastric cancer cells and induced apoptosis. The combination therapy of liquiritin and DPP significantly increased apoptosis and autophagy rates *in vitro* and *in vivo* by enhancing cleavage of caspase-8/-9/-3 and PARP and upregulating LC3B and Beclin-1 [91]. The combined application of liquiritin and TRAIL synergistically impeded the growth and proliferation of gastric cancer cells *in vitro* and the xenograft growth in nude mice through caspase activation [97]. Thus, liquiritin can significantly augment the therapeutic effects of other anticancer drugs and should be considered as an adjuvant in the treatment of human gastric cancer.

#### 3.1.4. Breast Cancer

Breast carcinoma is the most frequently diagnosed malignancy in women worldwide and is associated with high morbidity and mortality. The majority of breast-cancer-related deaths have been attributed to the metastasis of tumor cells to distant tissues, such as the brain or bone [98]. Licorice flavonoids including isoliquiritigenin [99], licochalcone A [100], and licochalcone E [101] can deter breast cancer progression through different mechanisms and signaling networks. Isoliquiritigenin inhibited the transcription and enzymatic activity of aromatase CYP19 that is involved in the synthesis of estrogen, which increases the risk of breast cancer [99]. It also inhibited the growth of MDA-MB-231 and MCF-7 cells by blocking the arachidonic acid (AA) metabolic network, which plays a crucial role in the growth of breast tumors [102]. In addition, isoliquiritigenin also inhibits key mediators and enzymes involved in breast carcinoma invasion and metastasis, such as VEGF, MMP-9, MMP-2, and hypoxia-inducible factor-1*α* (HIF-1*α*) [103]. Glabridin attenuated the cancer stem cells (CSCs) like properties of breast carcinoma cells, which is likely the major underlying cause of breast cancer metastasis and recurrence, by inhibiting the miR-148a/transforming growth factor-beta (TGF-*β*) drosophila mothers against decapentaplegic protein 2 (SMAD2) pathway both *in vitro* and *in vivo* [104]. Finally, licochalcone A displays proapoptotic and antiproliferative effects in breast cancer cells via modulation of transcription factor Sp1 (Sp1) and apoptosis-related proteins [100]. It also suppressed the proliferation, migration, and invasion of MDA-MB-231 breast carcinoma cells by increasing ROS production that triggered apoptosis and by regulating epithelial-mesenchymal transition factors like E-cadherin and vimentin [105].

#### 3.1.5. Other Tumors

The antitumor properties of licorice flavonoids have also been reported for the cancers of the colon [75], oral/esophageal squamous epithelium [106, 107], prostate [108], bladder [109], ovary [110], cervix, uterus [111], glioma [112], melanoma [113], uterine leiomyoma [114], and pleural mesothelioma [115]. The effects of flavonoid compounds against these tumors are summarized in Table 3.

### 3.2. Mechanism Underlying Anticancer Effect

**Figure 4:**
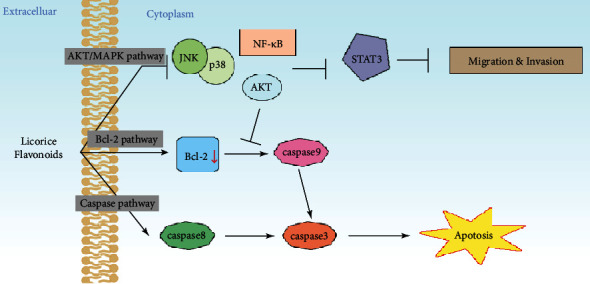
The main antitumor signaling pathway of licorice flavonoids.

Phytochemicals and other natural products can effectively inhibit the growth of tumor cells, augment the antitumor immune responses, and alleviate the side effects of radiotherapy. Several studies have shown that plant-derived bioactive compounds target pathways involved in tumor cell proliferation, differentiation, and metastasis, induce apoptosis, inhibit extracellular matrix enzymes, modulate the expression of transcription factors, and inhibit neoangiogenesis. In addition, several phytochemicals can promote the survival and expansion of antitumor immune cells and reverse the immunosuppressive tumor microenvironment [130]. Licorice flavonoids typically target the MAPK/JNK and PI3K/AKT pathways and also directly regulate the expression of genes involved in metastasis and apoptosis, as shown in Figure 4.

#### 3.2.1. MAPK/JNK Signaling Pathway

ERK, p38, and JNK are the key mediators of the MAPK signaling pathway in mammalian cells [131]. JNK contains a dual phosphorylated functional region that can bind to the N-terminal activation region of c-Jun and phosphorylate the serine residues at positions 63 and 73 [132]. It is activated by different stress-related stimuli and relays the signals through multiple pathways that regulate cancer genesis and progression [133]. Glabridin inhibited the proliferation of human liver cancer and oral cancer cells and induced apoptosis *via* the p38 MAPK and JNK1/2 pathways [76, 121]. Licochalcone A inhibited hepatocellular cell migration and invasion by downregulating uPA expression and activity through the inhibition of NF-*κ*B nuclear translocation and transcription of its downstream targets and that of the MKK4/JNK signaling pathway as well [134]. The antihepatocellular carcinoma effect of liquiritigenin has been attributed to MAPK inactivation, increased phosphorylation of JNK and p38, reduced expression of B-cell lymphoma-extra large (Bcl-xL) and Bcl-2, suppression of ERK, and decreased nuclear translocation of phosphorylated ERKs [83]. Licochalcone C induced apoptosis in human esophageal squamous cell carcinoma cells *via* ER stress response and ROS generation, which triggered mitochondrial dysfunction via JNK/p38 MAPK pathway activation [120].

#### 3.2.2. PI3K/AKT Pathway

The PI3K/AKT pathway is frequently dysregulated in multiple tumor cells [135]. It consists of several bifurcating and converging kinase cascades and is therefore a highly attractive therapeutic target [135, 136]. Several studies have shown that licorice flavonoids exert their antitumor effects by suppressing the PI3K/AKT pathway. Isoliquiritigenin not only inhibited cell cycle transition, proliferation, and migration of Hep3B cells but also inactivated the PI3K/AKT pathway in human breast tumor cells that resulted in growth retardation and apoptosis [78, 102]. Licochalcone A suppressed glycolysis and induced apoptosis in gastric cancer cells by inhibiting hexokinase 2 (HK2) and the AKT signaling pathway [95]. It also induced apoptosis in breast cancer cells and mitigated their migration and invasion by inhibiting Akt phosphorylation [105] and exhibited a proapoptotic effect in the BCC-823 gastric cancer cells through the PI3K/AKT-mediated pathway [109]. Liquiritigenin exerted significant inhibitory effects on the invasiveness and epithelial-mesenchymal transition of colorectal cancer cells by downregulating runt-related transcription factor 2 (Runx2) and inactivating the PI3K/AKT signaling pathway [117].

#### 3.2.3. Induction of Apoptosis in Tumor Cells

Apoptosis is a form of genetically programmed cell death characterized by membrane blebbing, cell shrinkage, and chromosomal DNA fragmentation. Most chemotherapeutic drugs and phytochemicals inhibit tumor growth by inducing the apoptotic cascade in cancer cells by targeting the enzymes, genes, and cytokines [137]. There are two major apoptotic pathways in the eukaryotic cell: the intrinsic mitochondrial-dependent pathway and the extrinsic death receptor-mediated pathway involving caspase activation [138]. Bax and Bak are members of the Bcl-2 family and the core regulators of the intrinsic apoptotic pathway. They are activated and oligomerized in the outer mitochondrial membrane and induce membrane depolarization under apoptotic stimuli [139]. Cysteine aspartic proteases or caspases are involved in inflammation, programmed cell death, and immune disorders [138].

Licochalcone A can induce apoptosis in the human hepatoma [80], lung cancer [86], osteosarcoma [129], bladder cancer [125], and prostate cancer [123] cells. It triggered the apoptotic cascade in HepG2 cells and human bladder cancer cells by upregulating Bcl-2, Bax, caspase-3, and caspase-8. It also decreased the expression levels of Bcl-2 and Bcl-xL in lung cancer cell lines, resulting in apoptosis. The cytotoxic effects of licochalcone A against human osteosarcoma cells and LNCaP prostate cancer cells are also mediated through the intrinsic apoptotic pathway and caspase-dependent cell death. Liquiritin triggered apoptosis in gastric cancer cells via both the intrinsic Bcl-2/Bax and extrinsic Fas-associated protein with death domain (FADD) regulated pathways, which culminated in cleavage of caspase-8 and caspase-9 [111]. It also induced apoptosis and autophagy in cancer cells when used in combination with cisplatin by enhancing caspase-8/-9/-3 and PARP cleavage [91].

## 4. Antioxidation

### 4.1. Antioxidant Effect

The antioxidant capacity of flavonoids is related to the molecular structure, associating with the position and the total number of -OH groups, conjugation and resonance effects, modification of the surrounding environment of thermodynamically favorable antioxidant sites, and the particular antioxidant mechanism of the compound [140].

Phenolic compounds derived from the roots and stolons of *G. glabra* exhibited considerable antioxidant action, as measured by the peroxynitrite assay, of which isoliquiritigenin, hispaglabridin B, and paratocarpin were the most potent antioxidants [141]. Liu et al. have reported that licorice extract (20, 40, and 60 mg/kg) containing licochalcone A, licoisoflavone, isolicocflavonol, and glycyrol reduced paraquat-induced oxidative stress in lung tissues by downregulating MDA level and increasing the SOD activity [142]. Polyphenols extracted from *Glycyrrhiza* also reduced the serum levels of total cholesterol, triglycerides, LDL cholesterol (LDL-C), and very-LDL-C by directly suppressing cholesterol biosynthesis and by indirectly eliminating free radicals and lowering LDL oxidation [143]. Additionally, flavonoid fraction from *G. glabra* showed remarkable antioxidant activity manifested by assays of low IC_50_ values in DPPH (20.9 mg/mL), NO radical scavenging (195.2 mg/mL), and hydrogen peroxide scavenging capacity (3.4 mg/mL) [144].

Furthermore, licochalcone B and licochalcone A significantly inhibited lipid peroxidation in rat liver microsomes and restrained LPS-induced ROS production in RAW 264.7 cells [32]. Glabridin reduced low-density lipoprotein (LDL) oxidation *in vitro* and *in vivo* [145]. Isoliquiritin exhibited a protective effect on neurodegenerative disorders through oxidative stress downregulation, intracellular [Ca^2+^]i overloading inhibition, and the mitochondrial apoptotic pathways suppression [146]. In addition, liquiritin alleviated cerebral ischemia/reperfusion injury in mice, as indicated by decreased infarct volume and less neurological deficit, through antioxidant and antiapoptosis mechanisms. It reduced the levels of malondialdehyde (MDA) and carbonyl, increased the ratio of glutathione (GSH/GSSG), and significantly decreased the percentage of apoptotic cells in the infarct region [147].

Other relevant reports with regard to antioxidation of licorice flavonoids and the specific structures are listed in Table 4.

### 4.2. Antioxidant Mechanism

Nrf2 controls the expression of antioxidant enzymes in management of oxidant stress [154]. Flavonoids derived from licorice exhibited reliable antioxidant activity through the regulation of Nrf2 protein expression. Isoliquiritigenin exerted its antioxidant effects by upregulating the transcription factors SKN-1/Nrf2 and DAF-16/FOXO, which activated genes involved in the antioxidant responses [151]. It also inhibited cigarette-smoke-induced oxidative stress in COPD by reversing MPO activity and decreasing MDA levels, upregulating Nrf2, and downregulating NF-*κ*B [64]. Isoliquiritigenin notably activated AMPK/Nrf2/ARE signaling and exhibited ROS producing inhibition in peritoneal macrophages of wild-type mice but not in Nrf2^−/−^ mice, illustrating that the antioxidative capacity of isoliquiritigenin relied on Nrf2 activation [148]. In addition, licochalcone A prevented ROS-driven oxidative stress in primary human fibroblasts *in vitro* by activating the cytoprotective phase II enzymes and stimulating the antioxidant transcription factor Nrf2 [156]. Glabridin displayed the antioxidant defense mechanism of liver *via* upregulating Nrf2 protein expression to lower the ROS formation and ameliorate oxidative stress exerting the hepatoprotective effect against MTX [154].

Furthermore, ultraweak photon emission analysis revealed significantly lower ultraviolet A (UV-A) stimulated luminescence *in vivo* following treatment with licochalcone A-rich licorice extract, which is indicative of lower oxidation [156]. Liquiritin abrogated oxidative injury in B65 neuroblastoma cells by increasing the expression of glucose-6-phosphate dehydrogenase in a dose-dependent manner [157]. Liquiritigenin, as an AMPK activator, protected hepatocytes against oxidant hepatic injury and mitochondrial dysfunction caused by nutrition deficiency which was attributed to LKB1-AMPK pathway activation and FXR induction [152]. Glabridin inhibits LDL oxidation by its direct antioxidant activity as well as by the removal of oxidized LDL through its paraoxonase activity [158]. Further research is needed to elucidate the exact mechanisms, as well as the structure-bioactivity relationship of licorice flavonoids to expand their applications as antioxidants.

## 5. Antibacterial, Antiviral, and Antiprotozoan Activity

Viral and other microbial infections play a critical role in many prevalent diseases, especially in developing countries [6]. Natural bioactive flavonoids derived from medicinal herbs and plants have been widely demonstrated to have antibacterial, antiviral, and antiprotozoan activity and can enhance the protective immune systems of human [159]. It is important to develop safe and effective antibacterial or antiviral agents, and licorice flavonoids have attracted much attention due to their excellent activity [6].

### 5.1. Effect on Diverse Microorganisms

Isoflavonoids such as 6,8-diisoprenyl-5,7,4′-thrihhydroxyisoflavone effectively inhibited the Gram-positive bacteria *Streptococcus mutans*, while gancaonin G displayed moderate antimicrobial activity [160]. Flavonoids from licorice, including glabrol, licochalcone A, licochalcone C, and licochalcone E, showed a favorable potential on Methicillin-resistant *Staphylococcus aureus* (MRSA) with low cytotoxicity for mammalian cells [161]. The flavonoid-rich fraction of the aqueous *Glycyrrhiza* extract has a potent anti-herpes-simplex-virus (HSV) activity; in addition, liquiritin, apioside, isoliquiritin apioside, lucurzid, and isoliquiritin have also been reported to be effective against HSV [162]. Licochalcone A displayed *in vitro* schistosomicidal effect on *Schistosoma mansoni* adult worms by affording lethal concentration for LC_50_ of 9.52 ± 0.9 and 9.12 ± 1.1 *μ*M against male and female adult worms, respectively, and it reduced the total number of *S. mansoni* eggs and impeded eggs produced by *S. mansoni* adult worms [163].

The studies associated with the effects of licorice flavonoids and their active constituents on bacteria, viruses, and protozoa are summarized in Table 5.

### 5.2. Mechanism Underlying Antimicrobial Action

In terms of mechanism, glabrol rapidly disrupted the proton movie force and membrane permeability of *S. aureus* possibly through binding to peptidoglycan, phosphatidylglycerol, and cardiolipin [161]. The flavonoid-rich extract of *G. glabra* inhibited *Helicobacter pylori* by downregulating DNA gyrase, dihydrofolate reductase, and protein synthesis [167]. The virulence of *Acinetobacter baumannii* was attenuated by the flavonoid-rich quorum quenching fraction of *G. glabra* via downregulation of autoinducer synthase and *abaI* expression [168]. Besides, liquiritin induced autophagy, apoptosis, and reduction of intracellular Ca^2+^ content of *Phytophthora capsici* and inhibited *P. capsici* pathogenicity *via* reducing PcCRN4 and Pc76RTF expressions as well as stimulating the plant defense which was reflected in the activated transcriptional expression of defense-related genes CaPR1, CaDEF1, and CaSAR82 and the increased antioxidant enzyme activity [175].

The *G. inflata*-derived chalcones inactivated the influenza A virus by inhibiting neuraminidase A [176]. Licocoumarone, licoflavonol, glyasperin D, and 2′-methoxyisoliquiritigenin exhibited antirotavirus activity, especially against G5P [7] and G8P [7] by suppressing both viral absorption and replication [177]. HSV-1 infection was suppressed by the quercetins extracted from *G. uralensis* via the inhibition of TLR-3, the inflammatory transcriptional factor NF-*κ*B, and interferon regulatory factor 3 (IRF3) [178].

Additionally, licochalcone A induced the death of adult *Schistosoma mansoni* by blocking superoxide dismutase activity, which increased the production of superoxide and other free radicals that directly damaged the worm tegument and membranes [163].

## 6. Antidiabetic Effect

### 6.1. Effect on Diabetes and Its Complications

Diabetes is a metabolic disease characterized by high blood sugar levels over a prolonged period. Clinical studies show that diabetes increases the risk of several complications such as renal damage, cataract, glaucoma, neuropathy, ischemic stroke, and gangrene among others. Many researchers have turned to discovering new drugs from natural products or traditional Chinese medicine owing to the specific toxic side effects of medications and insulin resistance [9].

Licorice flavonoids have shown significant antidiabetic effects. For instance, the ethanol extract of *G. glabra* can alleviate chronic hyperglycemia and diabetic nephropathy; in addition, the ethanol extract of *G. uralensis* inhibited the activity of liver microsomal diacylglycerol acyltransferase in obese and diabetic rats, and that of *G. inflata* was effective against diabetic nephropathy and diabetes-related vascular complications and endothelial dysfunction [182]. The flavonoid oil and ethanol extracts of licorice showed hypoglycemic and abdominal lipid-lowering effects in obese diabetic KK-A^y^ mice, which is clinically significant, since type 2 diabetes, hyperglycemia, obesity, and abdominal adiposity always develop simultaneously [183]. Moreover, licorice flavonoid oil exhibited therapeutic effects against diabetes and hyperglycemia in the KK-A^y^ mice by regulating glucose metabolism through AMPK pathway and the insulin levels in skeletal muscle [184]. In addition, licochalcone E promoted blood glucose elimination in hyperglycemic zebrafish, which restored calcium metabolism and impeded the generation of advanced glycation end-products (AGEs) [185]. Other studies on the antidiabetic effects of licorice flavonoids and their active components are summarized in Table 6.

### 6.2. Antidiabetic Mechanism

Protein tyrosine phosphatase 1B (PTP1B), a major negative regulator of the insulin-signaling cascade, is a promising therapeutic target for diabetes. Licorice flavonoids, including glycybenzofuran, licocoumarone, glycyrrhisoflavone, glisoflavone, isoliquiritigenin, licoflavone A, apigenin, glycycoumarin, and isoglycycoumarin [190], inhibited PTP1B activity with varying affinities and activated the insulin-signaling pathway [191]. Isoliquiritigenin blocked mesangial proliferation and matrix deposition during diabetic nephropathy by inactivating TGF-*β* RI and TGF-*β* RII and inhibiting the downstream SMAD signaling pathway [187]. STZ-induced diabetes was also alleviated by isoliquiritigenin via attenuation of oxidative stress, inflammation, and endothelial damage, downregulation of miR-195, restoration of retinal SIRT-1 levels, and tissue structure [150]. In addition, liquiritigenin significantly decreased high-fructose-diet-stimulated cardiac injury by mitigating tissue inflammation and fibrosis via suppression of the NF-*κ*B signaling pathway [189].

## 7. Treatment of Gastrointestinal Diseases

### 7.1. Gastrointestinal Protective Effect

The gastrointestinal tract of almost 50% of the global population is colonized by *H. pylori*, which is one of the most frequent causes of peptic ulcers [192]. Flavonoids of *Glycyrrhiza*, including licochalcone A (*G. inflata*), glabridin and glabrene (*G. glabra*), licoricidin, and licoisoflavone B (*G. uralensis*), inhibited the growth of *H. pylori in vitro* [193]. *GutGard*®, a flavonoid-rich fraction of *G. glabra*, inhibited *H. pylori* growth as per the microbroth dilution and agar dilution methods, with glabridin exhibiting the potent antibacterial effect [167]. Another study showed a special licorice extract (s-lico) that lowered the content of glycyrrhizin while enhancing the ratio of licochalcone A significantly inhibited *H. pylori*-induced gastritis as well tumorigenesis *in vivo* [194]. Apart from *H. pylori* infection, nonsteroid anti-inflammatory drugs (NSAIDs) abuse, irregular diet, excessive alcohol consumption, and smoking may also induce gastric or duodenal ulcers [192]. The hydroalcoholic extract of *G. glabra* significantly ameliorated HCL/ethanol-induced ulcer and also exerted a therapeutic effect on ulcers caused by indomethacin [195]. Isoliquiritigenin inhibited the occurrence of indomethacin-stimulated gastric ulcers through a favorable stomach distribution, ameliorated the gastric and hemorrhagic lesion sizes, and increased gastric mucus secretion as indicated by stronger Alcian blue staining intensity [196].

Both glabridin and licochalcone A have demonstrated therapeutic effects against inflammatory bowel disease (IBD). Gladridin mitigated the inflammation and colon damage in DSS-induced ulcerative colitis and improved hypertrophy and edema in the serosa and muscularis with lymphoid follicles hyperplasia [53]. Moreover, licochalcone A effectively alleviated colitis induced by DSS by reducing colon length, MPO activity, proinflammatory cytokine levels, and oxidative stress [197].

### 7.2. Gastrointestinal Protective Mechanism

The therapeutic effects of licorice flavonoids against gastric ulcer or gastritis induced by *H. pylori* may relate to restrain ^35^S methionine incorporating into *H. pylori* ATCC 700392 strain and inhibition of DNA gyrase and dihydrofolate reductase [167]. In addition, licorice flavonoids reversed the expressions of iNOS, COX-2, IL-8, and VEGF increased by *H. pylori* infection and significantly improved *H. pylori*- or hypoxia-induced angiogenesis [194]. Licorice flavonoids also alleviated NSAIDs- or chemical-induced gastric damage by regulating the content of small molecules metabolites like arachidonic acid, histamine, sphingosine-1-phosphate (S1P), tryptophan, and so forth, which mitigated inflammation and amino acid metabolism and increased gastric mucosal defensive factors [17, 195, 196]. Furthermore, glabridin reduced TNF-*α* levels and the expressions of iNOS and MPO genes in the colon tissues in a rat model of colitis, which was accompanied by a decrease in NO and an increase in cAMP levels [53]. Licochalcone A alleviated DSS-induced ulcerative colitis by restoring the expression levels of I*κ*B kinase *α* (IKK*α*), p65 NF-*κ*B, and p-I*κ*B, upregulating *γ*-glutamyl cysteine ligase (GCL), Nrf2, and HO-1, and downregulating Kelch-like ECH-associated protein 1 (Keap1) in the colonic tissues [197].

## 8. Effect on Skin Disorders

### 8.1. Skin Protective Effect

Cho et al. found that topical application of licochalcone E decreased ear edema and thickness, epidermal detachment, and focal microabscesses in the oxazolone-induced chronic dermatitis mouse model in a dose-dependent manner. Mechanistically, licochalcone E decreased the expression of IL-12p40 and IFN-*γ* in the affected mice, which mitigated other symptoms of inflammation as well [62]. Atopic dermatitis (AD) is a common chronic inflammatory skin disorder that is currently treated with steroids, which have severe side effects in most of the patients. Isoliquiritigenin ameliorated AD-like symptoms in a mouse model, reduced scratching behavior and the severity of skin lesions [40], and can be considered as a safer alternative to steroid therapy. Furthermore, Wu et al. have reported that isoliquiritigenin ameliorated the inflammatory process in various psoriasis models, including VEGF transgenic mouse, the imiquimod-induced psoriasis-like mouse, and the human keratinocytes HaCaT and NHEK cell [57].

The therapeutic effects of topically applied licorice flavonoids on sensitive skin or inflammatory dermatosis have been demonstrated in recent clinical trials [198, 199]. The incorporation of licochalcone A in the skin care regimens was well tolerated by the sensitive skin of rosacea patients. After 8 weeks of treatment, redness was significantly reduced and other signs of rosacea were also neutralized. A randomized, prospective, investigator-blinded study showed that a moisturizer formulation containing licochalcone A improved facial dermatitis, erythema, and skin hydration compared to 0.02% triamcinolone acetonide [200].

Tyrosinase is a kind of oxidase, which is the rate-limiting enzyme that controls the production of melanin in human body. Once melanin overproduces, it will lead to a variety of skin diseases [201]. Licorice flavonoid is a natural skin-lighting agent, especially the component glabridin which is regarded as the “whitening gold,” and it exhibited reversibly inhibition of tyrosinase in a noncompetitive manner by a multiphase kinetic process with IC_50_ of 0.43 *μ*mol/L; besides, it bound to tyrosinase with a static process and a stable complex of glabridin-tyrosinase may be generated [201]. In general, glabridin is consumed as a constituent of licorice extract; for instance, glabridin-40, one of glabridin-rich licorice extracts, is widely applied in cosmetic products as a skin-whitening and is used as an antioxidant and anti-inflammatory agent [202].

### 8.2. Skin Protective Mechanism

Licochalcone E improved inflammatory skin disorders by inhibiting NF-*κ*B activation and nuclear translocation through I*κ*B*α* dephosphorylation [62]. Furthermore, licochalcone E exhibited anti-inflammatory effects on mouse skin and murine macrophages by suppressing AP-1 and NF-*κ*B transcriptional activity, inactivation of AKT and MAPK, and downregulation of iNOS, COX-2, and proinflammatory cytokines [203]. Isoliquiritigenin ameliorated DNCB-stimulated atopic dermatitis through decreasing Th2 and IgE cytokines, inhibiting proinflammatory cytokines, eliminating p38-*α* and ERK activation, and upregulating CD54 and CD86 in human monocyte model THP-1 [40]. Isoliquiritigenin suppressed psoriasis-like symptoms through the inhibition of NF-*κ*B activity which consequently led to the less expressions of proinflammation cytokines IL-6 and IL-8 [57]. The skin-lighting mechanism of glabridin has been deduced by molecular docking experiment where glabridin may interact between the hydroxyl group of glabridin and the active site residues (mainly His-85) attributing to a type of stereospecific blockade effect or deformation of the catalytic core domain, which resulted in suppressing the oxidant activity of tyrosinase to substrate L-3,4-dihydroxyphenylalanine (L-DOPA) [201].

## 9. Effect on Obesity

### 9.1. Antiobesity Effect

Obesity is characterized by the excess accumulation of lipid metabolites in adipose and nonadipose tissues [204] and is currently a pressing health issue worldwide due to its association with a high risk of cardiovascular diseases, type 2 diabetes, hypertension, and cancer [205]. Glabridin reduced the body weight of obese mice by decreasing food intake and increasing energy expenditure [206]. In addition, licorice flavonoid oil prevented and regulated diet-induced obesity and total body fat in human subjects by restoring the expression levels of the related lipid metabolism [207]. White adipose tissues are energy depots, whereas brown adipose tissues convert energy to heat *via* thermogenesis and improve triglyceride clearance and glucose metabolism [208, 209]. Inducible brown adipocytes can be developed in white adipose tissues through the browning process, which is a viable strategy for treating obesity and its complications [210]. Licochalcone E has demonstrated an inhibitory effect in early adipogenesis, as indicated by enhanced adipocyte differentiation, reduction in adipocyte size, and increased population of small adipocytes in white adipose tissues [211].

### 9.2. Antiobesity Mechanism

Glabridin inhibited the expression of lipogenic genes such as fatty acid synthase (FAS), sterol regulatory element-binding protein-1c (SREBP-1c), stearoyl-CoA desaturase-1 (SCD-1), and acetyl-CoA carboxylase (ACC) in the white adipose tissues and liver of several animal models of obesity and upregulated fatty acid oxidation genes in the muscle, eventually leading to a decrease in body weight and fat cell size through AMPK activation [206]. Licochalcone E upregulated PPAR*γ* by activating the AKT pathway and facilitated adipocyte differentiation and increased the number of small adipocytes, thereby ameliorating hyperglycemia and hyperlipidemia under diabetic conditions [211]. Licochalcone A activated the sirt-1/AMPK pathway to enhance lipolysis and *β*-oxidation and reduce fatty acid chain synthesis [210]. Moreover, it upregulated the expression of brown fat markers including PR domain containing 16 (PRDM16), uncoupling protein 1 (UCP1), and PPAR*γ* coactivator-1 (PGC-1*α*), which reduced obesity and restored metabolic homeostasis by altering brown fat phenotype [212].

## 10. Conclusion

Licorice is the most frequently prescribed herbal medicine in China, and it consists of abundant flavonoid components with a multitude of pharmacological effects. In recent years, licorice flavonoids have been isolated and characterized, and the mechanisms underlying their pharmacological effects have been elucidated. The therapeutic effects of licorice flavonoids against gastrointestinal and skin diseases have even been clinically tested. Other biological properties of licorice flavonoids including the antiviral, antibacterial, antidiabetic, antiasthma, and anticancer effects have been demonstrated at the cellular and animal level and need to be validated in clinical trials. Furthermore, the molecular mechanisms underlying the pharmacological action of licorice flavonoids need to be lucubrated due to the complex interaction between diverse components and organisms. In this review, the pharmacological properties of licorice flavonoids in various pathological conditions and the possible mechanisms of action were detailly summarized. The article expands the application of licorice flavonoids, which provides a preference for further research on material basis, bioactivity, and mechanism of licorice flavonoids in the future.

## Figures and Tables

**Table 1:** The anti-inflammatory properties of licorice flavonoids *in vitro*.

Compounds	Dose	Inflammation	Cell line/tissue	Inhibition	References
Licochalcone A	5–20 *μ*M	LPS-induced inflammatory reactions	RAW 264.7 cell	Reduced the concentration of TNF-*α*, IL-6, and IL-1*β*	[35]
	3 and 10 *μ*M	LPS-induced inflammatory reactions	RAW 264.7 cell	Dose-dependently inhibited LPS-induced ROS production and reduced the generation of NO, IL-6, and PGE2	[32]
	15 nM	IL-1*β*-stimulated inflammation	Normal human dermal fibroblasts	Exhibited the 50% inhibition of COX-2-dependent PGE2 production	[36]
	5–20 *μ*M	IL-1*β*/TNF-*α*-stimulated inflammation	Primary chondrocytes	Inhibited PGE2, NO, iNOS, COX-2, matrix metalloproteinase-1 (MMP-1), MMP-13, and MMP-3 production in chondrocytes	[37]
Licochalcone C	50 *μ*M	LPS- (10 *μ*g/mL) and interferon-*γ* (IFN-*γ*) (20 ng/mL) stimulated inflammation in THP-1 cell	Human myeloid leukemia mononuclear cell (THP-1)	Attenuated inflammatory response by diminishing the expression and activity of iNOS, by modulating extracellular O_2_^−^ generation and by restraining the activity of superoxide dismutase (SOD), catalase (CAT), and glutathione peroxidase (GPx)	[38]
Isoliquiritigenin	2.5–10 *μ*g/ml	LPS- (0.1 *μ*g/ml) induced proinflammatory mediators production	J774A.1 murine macrophage cell line	Inhibited NO, IL-1*β*, and IL-6 production dose-dependently	[39]
	10 *μ*M	2,4-Dinitrochlorobenzene (DNCB) induced atopic dermatitis	THP-1 cell line	Suppressed the differentiation of CD54 and CD86 and restrained the activation of extracellular signal-regulated kinase (ERK) and p38-*α* mitogen-activated protein kinase (p38-*α*)	[40]
Glabridin	5–10 *μ*g/ml	LPS (0.1 *μ*g/ml) induced proinflammatory mediators production	J774A.1 murine macrophage cell line	Moderate inhibition in NO levels with a maximum inhibition of 33% at the highest tested concentration	[39]
	5–20 *μ*M	LPS (1 *μ*g/mL) stimulated inflammation	HaCaT cell line	At 20 *μ*M, the inhibition reached 47%, 53%, and 68% for IL-1*β*, IL-6, and p65, respectively; and the suppression reached 53%, 55%, and 45% for IL-17A, IL-22, and IL-23	[41]
	1–10 *μ*M	TNF-*α* (10^−10^ M) induced increase of PGE2 and NO in osteoblasts	MC3T3-E1 cells	The release of PGE2 and the increase of NO in osteoblasts were decreased significantly	[42]
Liquiritin	50 and 100 *μ*M	LPS (100 ng/mL) stimulated microglial cell model	Murine BV2 cell line	Inhibited the increase of NO and proinflammatory mediators iNOS, COX-2, IL-1*β*, TNF-*α*, and IL-6	[43]
Liquiritigenin	50 and 100 *μ*M	LPS- (100 ng/mL) induced microglial cell model	Murine BV2 cell line	Suppressed the augment of NO and proinflammatory mediators COX-2, iNOS, IL-1*β*, IL-6, and TNF-*α*	[43]
	20 and 40 *μ*M	IL-1*β* (10 ng/ml) induced inflammation	Chondrocytes from 1-week-old Sprague-Dawley rats	Inhibited the IL-1*β*-induced expression of NO and PGE2	[44]
Licoricidin	0.5–1 *μ*g/ml	LPS-stimulated secretion of cytokines and MMPs by human monocyte-derived macrophages	Human monoblastic leukemia cell line	Inhibited the secretion of IL-6, chemokine ligand 5, and MMP-7, MMP-8, and MMP-9	[45]

**Table 2:** The anti-inflammatory properties of licorice flavonoids *in vivo*.

Compounds	Dose and administration	Inflammation tissues/diseases	Animal	Outcomes	References
Total flavonoids	50 and 100 mg/kg once a day for 10 weeks (i.g.)	Azoxymethane/dextran sulfate sodium (AOM/DSS) stimulated colonic inflammation	Female C57BL/6 mice weighing 16–18 g	Greatly suppressed colitis and colorectal tumorigenesis by suppressing the production of inflammatory cytokines and phosphorylation	[46]
	1.56 g crude drugs per kilogram per day for 3 weeks (i.g.)	Arthritis induced by injection of complete Freund's adjuvant (CFA)	Sprague-Dawley (SD) rats (200 ± 20 g)	Exhibited therapeutic effects on acute inflammation, chronic inflammation, and inflammatory pain and reduced IL-1*β* and TNF-*α* in plasma level	[47]
	3–30 mg/kg (i.g.) for 5 times with an interval of 6 h before LPS instillation and for 2 times with an interval of 8 h after LPS instillation	LPS (2 mg/ml) induced acute inflammation of lung	ICR mice	Significantly attenuated LPS-induced pulmonary inflammation by suppressing inflammatory cells infiltration and inflammatory mediator release and reduced neutrophil-mediated oxidative injury	[30]
	500 and 250 mg/kg (i.g.) with 40 min before *carrageenan injection*	1% (w/v) *carrageenan-induced paw edema*	SD rats (180–220 g)	Significantly ameliorated edema and reduced the expression of TNF-*α*, IL-1*β*, and iNOS at a dose of 500 mg/kg	[29]
Licochalcone A	20, 40, and 80 mg/kg (i.p.) at 1 h prior to LPS administration/1 h after LPS challenge	LPS-induced lung injury/acute kidney	BALB/c mice/female C57BL/6 mice	Attenuated lung/kidney histopathologic changes and inhibited the production of TNF-*α* and IL-1*β* induced by LPS	[35, 48]
	50 mg/kg (i.p.) at 1 h before OVA challenges on days 25–27	Ovalbumin (OVA) stimulated inflammation on noninfectious asthma	Female BALB/c mice, weighing about 16–18 g	Inhibited T-helper type 2 cytokines like IL-4, IL-13, and IL-5 in bronchoalveolar lavage fluid and decreased serum levels of OVA-specific IgG and IgE	[49]
Isoliquiritigenin	20 mg/kg (i.p.) administered at 30 min, 12 h, and 24 h prior to LPS treatment/5–20 mg/kg given 1 h before LPS challenge	LPS-induced neuroinflammation/acute lung injury	Male Wistar rats/BALB/c mice	Reversed LPS-induced increase in expression of TNF-*α* and IL-1*β* and decreased NF-*κ*B activity	[50, 51]
	Treated with 1% isoliquiritigenin on dorsal skin daily from day 6 to day 18	Repetitive application of DNCB-induced atopic dermatitis-like skin lesion	6–8-week-old BALB/c mice	Suppressed the IgE and Th2 cytokines increase in blood and inhibited the expressions of IL-6, TNF-*α*, and IL-4 at the site of skin lesion	[40]
	Gavaged 7.5–75 mg/kg at 24 hours and 1 h prior to indomethacin challenge	Indomethacin (10 mg/kg) induced small intestinal damage	Wild-type male C57BL/6 mice (7-week-old)	Reversed indomethacin-induced increase in cleaved caspase-1 and mature IL-1*β* protein levels	[52]
Glabridin	Glabridin (10, 30, and 50 mg/kg/d) pretreated on shaved back for 7 days	Imiquimod-induced psoriasis-like inflammation	BALB/c mice averagely weighted 20–25 g	Significantly downregulated the mRNA expressions of IL-1*β*, IL-6, IL-17A, IL-22, IL-23, and p65	[41]
	Gavaged 10 or 50 mg/kg/d 1 week before colitis induction and parallel with DSS-feeding for 7 days	Dextran sulfate sodium (DSS, 5%) induced colonic inflammation	Adult male Wistar rats/six-week-old female BALB/c mice	Ameliorated the disruption of the colonic architecture and reduced myeloperoxidase (MPO) activity and production of inflammatory mediators in colon	[53, 54]

**Table 3:** Anticancer/tumor effects of licorice flavonoid and its active components in experimental models.

Cancer	Compounds	Dose and administration	Result	References
Colon	Isoangustone A	5–20 *μ*M incubated	Induced apoptosis in colorectal cancer cells	[116]
	Total flavonoids	Gavaged (50 and 100 mg/kg) once a day for 28 days	Restrained AOM/DSS-induced colitis-associated tumorigenesis, reduced activation of p53 and NF-*κ*B, and suppressed phosphorylated-Janus kinases 2 (p-JAK2) and phosphorylated-signal transducer and activator of transcription 3 (p-STAT3) production	[46]
	Liquiritigenin	50 and 100 *μ*M incubated	Exerted significant inhibitory effects on HCT116 colorectal cancer cell invasion and blocked the epithelial-mesenchymal transition (EMT) process	[117]
Oral/esophageal squamous	Licochalcone A	10–40 *μ*M incubated	Inhibited HN22 and HSC4 oral squamous cell carcinoma cells growth concentration- and time-dependently	[118]
	Licochalcone B	10–30 *μ*M incubated	Arrested cell cycle at G1 phase, significantly inhibited cell proliferation, and induced apoptosis in oral squamous cell carcinoma cells	[119]
	Licochalcone C	10–30 *μ*M incubated for 48 h	Significantly decreased cell viability of esophageal squamous cell carcinoma (ESCC) cells in a dose- and time-dependent manner	[120]
	Licochalcone H	10–30 *μ*M incubated	Induced cell cycle arrest and apoptosis, reduced cell activity, and colony-forming ability in HSC2 and HSC3 oral squamous cell carcinoma cells	[77]
	Glabridin	20–80 *μ*M incubated	Inhibited cell proliferation in human tongue squamous carcinoma cell lines (SCC-9 and SAS) and induced several features of apoptosis	[121]
	Isoliquiritigenin	25 and 50 *μ*M incubated	Induced cell cycle G2/M phase arrest, DNA damage, and apoptosis in oral squamous cell carcinoma cells	[122]
Prostate	Licochalcone A	6.5 and 12.5 *μ*M incubated	Induced caspase-dependent and autophagy-related cell death in LNCaP cells	[123]
	Isoliquiritigenin	25 and 50 *μ*M incubated	Suppressed cell proliferation, induced cell apoptosis, and arrested G2/M cell cycle in human prostate cancer PC-3 and 22RV1 cells	[124]
Bladder	Licochalcone A	10–40 *μ*M incubated	Exerted antiproliferative effect on human bladder cancer cells and induced G2/M cell cycle arrest and apoptotic cell death	[125]
Ovary	Isoliquiritigenin	5 and 10 *μ*M incubated	Inhibited epithelial-to-mesenchymal transition, migration, and invasion in SKOV3 and OVCAR5 ovarian cancer cells and extended the life span of animals bearing SKOV3/Luc cells consequently	[126]
Cervix uteri	Liquiritin	40–80 *μ*M incubated	Suppressed the migration, invasion, and cloning ability of cervical cancer cells and showed little cytotoxicity to human normal cells	[111]
Glioma	Licochalcone A	10–30 *μ*M incubated	Inhibited glioma cell growth in U87 glioma cell lines and U87 glioma cell xenograft male athymic mice	[127]
Melanoma	Isoliquiritigenin	20–80 *μ*M incubated	Effectively induced apoptosis and inhibited proliferation in mouse melanoma B16F10 cells	[128]
Uterine leiomyoma	Isoliquiritigenin	10–40 *μ*M incubated *in vivo*; 1 and 5 mg/ml (i.p.) three times a week for 9 weeks on ICR mice	Exerted inhibition of estrogen-induced uterine leiomyoma growth both *in vitro* and *in vivo*	[114]
Pleural mesothelioma	Licochalcone A	10–40 *μ*M incubated	Induced apoptosis through suppressing Sp1 expression in malignant pleural mesothelioma cell MSTO-211H and H28	[115]
Osteosarcoma	Licochalcone A	20–60 *μ*M incubated with human osteosarcoma cells; 10 mg/ml (i.p.) twice a week for 5 weeks on BALB/c nude mice	Inhibited cell proliferation and induced apoptosis in human osteosarcoma cells by reduction of cell viability, activation of caspases, and loss of mitochondrial membrane potentials	[129]

**Table 4:** The antioxidant properties of licorice flavonoids.

Compounds	Model	Dose and effects	References
Isoliquiritigenin	LPS-induced acute lung injury mice	Treatment with isoliquiritigenin (30 mg/kg) enhanced the production of ROS, MPO, and MDA, ameliorating low expression of GSH and SOD caused by LPS stimulation	[148]
	LPS-induced cognitive impairment rats	Isoliquiritigenin (20 mg/kg) pretreatment appeared antioxidant capacity through reversing the downregulation of SOD and GSH-PX activity and reducing the content of MDA	[149]
	Streptozotocin (STZ) induced diabetic retinopathy	Isoliquiritigenin (20 mg/kg/day) treatment markedly reduced diabetes-induced lipid peroxidation by 27.8%, upregulated retinal GSH 1.57-fold, and restored total retinal antioxidant capacity 2.15-fold	[150]
	*Caenorhabditis elegans*	Isoliquiritigenin (50 *μ*g/ml) reduced heat shock protein-16.2 (HSP-16.2) expression level by 30.8% under mild oxidative stress and increased the survival rate of *C. elegans* from 10.8% of control group to 97.4% under lethal oxidative stress	[151]
Liquiritigenin	Serum deprivation in HepG2, H4IIE, and AML12 cells induced oxidative stress	Mitochondrial dysfunction, oxidative stress like ROS formation, and resultant cell death caused by nutrition deprivation were prohibited by liquiritigenin (100 *μ*M) pretreatment	[152]
	Citrinin (CTN) induced, oxidative-stress-mediated disruption of embryonic development in mouse blastocysts	CTN-triggered ROS generation for sequent apoptosis and injury of blastocysts was restrained by the preincubation of liquiritigenin (20–40 *μ*M)	[153]
Glabridin	Methotrexate (MTX) triggered liver injury	Glabridin (20 or 40 mg/kg) lower oxidative stress stimulated by MTX through upregulation of MDA level, as well as reduction of GSH level and SOD activity	[154]
	Diabetic vascular complications mouse	Glabridin prevented the antiatherogenic capacity of paraoxonase 2 (PON2) by the interaction of glabridin-PON2 that protected PON2 from oxidation	[155]
Licochalcone A	HepG2 cell and L-02 cell	Licochalcone A inhibited peroxyl radical-induced oxidation of DCFH to DCF in HepG2 cells in a dose-dependent manner and upregulated protein expression of SOD1, CAT, and GPx1 at 2–8 *μ*g/ml	[74]

**Table 5:** The antibacterial, antiviral, and antiprotozoan effects of licorice flavonoids.

Effects	Compounds	Microorganism	Dose and effect	References
Antibacterial effects	1-Methoxyficifolinol, licorisoflavan A, and 6,8-diprenylgenistein	*Streptococcus mutans*	Showed bactericidal effects at the concentration of ≥4 *μ*g/ml	[164]
	Flavonoid of *G. uralensis* extracts	*Streptococcus mutans* and *Candida albicans*	The inhibition zones of *S. mutans* and *C. albicans* increased in order: 50 *μ*g/ml < 100 *μ*g/ml < 150 *μ*g/ml < 200 *μ*g/ml	[165]
	Licoricidin and glabridin	*Streptococcus mutans*	Licoricidin had an MIC of 6.25 *μ*g/mL and an MBC between 6.25 and 25 *μ*g/mL; glabridin showed an MIC from 6.25 to 12.5 *μ*g/mL and an MBC between 6.25 and 25 *μ*g/mL against one reference (ATCC 25175) and four clinical (12A, 33A, INB, and T8) strains of *S. mutans*	[166]
	Flavonoid-rich extract of *G. glabra*	*Helicobacter pylori*	At minimum inhibitory concentration (MIC) of 100 *μ*g/ml	[167]
	Flavonoids of *G. glabra*, namely, licoricone, glycyrin, and glyzarin	*Acinetobacter baumannii*	Significantly reduced quorum sensing regulated virulence factors of *A. baumannii* at 0.5 mg/ml	[168]
	Isoliquiritigenin and liquiritigenin	MRSA	MIC of both components exhibited significant anti-MRSA activity (50–100 *μ*g/ml) against clinical isolates of MRSA	[169]
	Licochalcone A	*Bacillus subtilis*	The vegetative cell growth of *B. subtilis* was inhibited in a concentration-dependent manner and was completely prevented by 3 *μ*g/ml	[170]
	Licochalcone A	*Candida albicans*	Reduced *C. albicans* biofilm growth at 625 *μ*M *in vitro*; and mice treated with licochalcone A exhibited a markable reduction in total photon flux and CFU/ml/mg of tongue tissue sample	[171]
	Nisin/glabridin, nisin/licoricidin, and nisin/licochalcone A	*Enterococcus faecalis*	Efficiently restrained the growth of *E. faecalis*, with MICs ranging from 6.25 to 25 *μ*g/mL	[172]
	6-Aldehydo-isoophiopogonone and liquiritigenin	Multidrug-resistant human bacterial *Staphylococcus aureus*	6-Aldehydo-isoophiopogonone and liquiritigenin showed activity against *S. aureus* with a zone inhibition of 10 ± 0.2 mm and 10 ± 0.3 mm	[173]
	Glabridin	Amphotericin B resistant *Candida albicans*	At an MIC of 31.25–250 *μ*g/mL	[174]
	Liquiritin	*Phytophthora capsici*	Suppressed the *P. capsici* mycelial growth with EC_50_ of 658.4 mg/L and caused *P. capsici* sporangia to shrink and collapse	[175]
Antiviral effects	Echinantin and isoliquiritigenin	Influenza A viruses	Showed strong inhibitory effects on various neuraminidases from influenza viral strains, H1N1, H9N2, novel H1N1 (WT), and oseltamivir-resistant novel H1N1 (H274Y) expressed in 293T cells	[176]
	Licocoumarone, glyasperin C, 2′-methoxyisoliquiritigenin, glycyrin, licoflavonol, and glyasperin D	Rotaviruses, specially G5P [7] and G8P [7]	The 50% effective inhibitory concentrations (EC_50_) of the six compounds were 18.7–69.5 *μ*M against G5P [7] and 14.7–88.1 *μ*M against G8P [7]	[177]
	Quercetin of *G. uralensis*	Herpes simplex virus-1 (HSV-1)	Showed 50% decrease for 10 *μ*g/ml quercetin and 90% decrease for 30 *μ*g/ml of quercetin in plaque formation in Vero cells when incubated with infected cell lysates treated with quercetin; dose-dependently suppressed HSV-1 infection in Raw 264.7 cells	[178]
	Kanzonol Y	Dengue virus (DENV)	Exhibited anti-dengue-virus activity due to the outstanding docking properties with DENV protease, DENV RNA-dependent RNA polymerase, and DENV envelope protein	[179]
	Isobavachalcone	Porcine reproductive and respiratory syndrome virus (PRRSV)	Had potential anti-PRRSV activity and inhibited PRRSV replication at the postentry stage of PRRSV infection	[180]
Antiprotozoan effects	Licochalcone A	Chloroquine-susceptible (3D7) and chloroquine-resistant (Ddz) strains of *Plasmodium falciparum*	Had potent antiplasmodial efficacy against chloroquine-susceptible (3D7) and chloroquine-resistant (Ddz) strains of *Plasmodium falciparum* in vitro	[181]

**Table 6:** The antihyperglycemic effects of licorice flavonoids.

Compounds	Dose and administration	Result	References
Isoangustone A	1–20 mmol/L incubated with human renal mesangial cells (HRMC) for three days	High glucose-inflammatory mesangial hyperplasia and matrix dilation were retarded by the accumulation of type IV collagen, and diabetes-related renal inflammation was reduced by attenuating inflammatory ICAM-1 expression and monocyte chemotactic protein-1 (MCP-1) production in the mesangium	[186]
Isoliquiritigenin	1–20 *μ*M incubated with HRMC for 3 days	Prevented mesangial fibrosis and glomerulosclerosis generating into renal failure and end-stage renal diseases through diminishing high glucose-related mesangial matrix accumulation	[187]
Glabridin	3T3‐L1 adipocytes incubated with 5–20 *μ*M of glabridin 1 hour before 2,3,7,8‐tetrachlorodibenzo‐p‐dioxin (TCDD, 10 nM) challenge for 3 days	Restored TCDD-descended insulin‐stimulated glucose uptake and production of glucose transporter 4 (GLUT4) and insulin receptor substrate 1 (IRS1)	[188]
Isoliquiritigenin	20 mg/kg/day to diabetic rats for 8 weeks	Ameliorated diabetes-induced retinal injury in STZ-induced diabetic rats	[150]
Liquiritigenin	4–16 mg/kg liquiritigenin-treated mice after fructose feeding	Reduced fructose-diet-induced lipid accumulation and cardiac fibrosis that exerted protective response in high-fructose-diet-triggered cardiac injury	[189]
